# Examining the Association of Body Mass Index and Complications When Including Sentinel Lymph Node Biopsy in Minimally Invasive Surgery for Endometrial Intraepithelial Neoplasia

**DOI:** 10.3390/cancers17081257

**Published:** 2025-04-08

**Authors:** Gabriel Levin, Pedro T. Ramirez, Jason D. Wright, Brian M. Slomovitz, Walter H. Gotlieb, Matthew T. Siedhoff, Kelly N. Wright, Raanan Meyer

**Affiliations:** 1 Division of Gynecologic Oncology, Jewish General Hospital, McGill University, Montreal, QC H3A 0G4, Canada; walter.gotlieb@mcgill.ca; 2 Department of Obstetrics and Gynecology, Houston, Methodist Hospital, Houston, TX 77030, USA; pramirez3@houstonmethodist.org; 3 Department of Gynecologic Oncology, Columbia University College of Physicians and Surgeons, New York, NY 10032, USA; jw2459@cumc.columbia.edu; 4 Herbert Irving Comprehensive Cancer Center, New York, NY 10032, USA; 5 New York-Presbyterian Hospital, New York, NY 10033, USA; 6 Mount Sinai Medical Center, Miami, FL 33140, USA; bslomovitz@gog.org; 7 Division of Minimally Invasive Gynecologic Surgery, Department of Obstetrics and Gynecology, Cedars Sinai Medical Center, Los Angeles, CA 90048, USA; matthew.siedhoff@cshs.org (M.T.S.); kelly.wright@cshs.org (K.N.W.); raananmeir@gmail.com (R.M.); 8 Faculty of Medicine, Tel-Aviv University, Tel-Aviv 6997801, Israel; 9 The Dr. Pinchas Bornstein Talpiot Medical Leadership Program, Sheba Medical Center, Tel Hashomer, Ramat-Gan 5262000, Israel

**Keywords:** body mass index, complications, endometrial cancer, minimally invasive surgery, sentinel lymph node biopsy

## Abstract

**Objective**: We aimed to study the association of patient’s body mass index (BMI) with postoperative complications in patients surgically treated for endometrial intraepithelial neoplasia, with and without sentinel lymph node biopsy. **Methods**: A cohort study using the prospective National Surgical Quality Improvement Program database. Women with endometrial intraepithelial neoplasia on postoperative pathology who underwent minimally invasive hysterectomy from January 2012 to December 2020 were included. The cohort was dichotomized based on the performance of sentinel lymph node biopsy. We analyzed postoperative complications based on the World Health Organization (WHO) categories of BMI. **Results**: A total of 4428 patients met the inclusion criteria. Of those, 584 (13.2%) had sentinel lymph node biopsy. Overall, 76.5% of patients (n = 3389) were obese (BMI > 30.0), with 1840 (41.6%) patients of BMI ≥ 40.0. The rate of any complications was 6.0% (n = 264), major complications 2.3% (n = 101), and minor complications 4.2% (n = 187). When comparing the rate of any complications between patients who had sentinel lymph node biopsy vs. those without a sentinel lymph node biopsy procedure, stratified by BMI category, there was no association between sentinel lymph node biopsy performance and any complications in any of the BMI categories. In a multivariable binary regression analysis, BMI and the performance of sentinel lymph node biopsy were not independently associated with any complication [adjusted odds ratio (aORs) 1.001, 95% confidence interval (CI) (0.98–1.01), and aORs 1.1, 95% CI (0.82–1.65), respectively]. In an analysis of the cohort of patients who underwent sentinel lymph node biopsy, there was no association between the rates of any major or minor complications with BMI categories or obesity. ROC analyses for the association between BMI and occurrence of any major or minor complications had a low performance. **Conclusions**: In minimally invasive surgery for endometrial intraepithelial neoplasia, there is no association between body mass index and increased risk for postoperative complications when performing hysterectomy with sentinel lymph node biopsy versus hysterectomy alone.

## Simple Summary

This study aimed to examine the relationship between body mass index (BMI) and postoperative complications in women who underwent minimally invasive hysterectomy for endometrial intraepithelial neoplasia, with or without sentinel lymph node biopsy. The cohort consisted of 4428 patients, with 584 undergoing sentinel lymph node biopsy. The majority (76.5%) were obese, and 41.6% had a BMI of 40 or higher. The overall complication rate was 6%, with no significant difference in complications between those with and without sentinel lymph node biopsy, regardless of BMI. The regression analysis showed that neither BMI nor sentinel lymph node biopsy was independently linked to complications. Additionally, for patients who underwent sentinel lymph node biopsy, BMI showed no association with any type of complication. This study concluded that in minimally invasive surgery for this condition, BMI does not correlate with an increased risk of postoperative complications, regardless of the procedure type.

## 1. Introduction

Endometrial intraepithelial neoplasia (EIN) is diagnosed in approximately 1% of premenopausal women with abnormal uterine bleeding [1] and in approximately 6% of postmenopausal women with uterine bleeding, thickened endometrium, and benign endometrial biopsy [2]. Up to 50% of patients with a preoperative diagnosis of EIN will be diagnosed with endometrial carcinoma on the final pathological specimen [3,4,5,6,7,8,9,10]. Though not explicitly supported by the American College of Obstetrics and Gynecology (ACOG) [6], there is a growing trend of including sentinel lymph node biopsy (SLNB) during surgery for EIN, including more than 25% of minimally invasive hysterectomies for EIN in 2020 in the US [11].

Obesity is an established risk factor for endometrial cancer, and its precursor, EIN [12,13,14,15]. It is estimated that by 2030, half of US adults will be obese and a quarter morbidly obese [16]. With similar trends in this obesity epidemic seen globally [17], it is important to improve our knowledge and understanding of the impact of body mass index (BMI) on the outcomes of surgical treatment for EIN. There is an association of BMI with postoperative complications following hysterectomy for endometrial cancer, with obese patients suffering from higher rates of postoperative surgical complications [18]. However, the literature regarding the association of BMI and surgical complications following minimally invasive hysterectomy for EIN is underreported. As there are concerns associated with SLNB’s technical complexity in obese patients, and the uncertain benefits it may provide for patients with EIN [19,20], it is imperative to study whether the performance of SLNB in EIN obese patient is associated with higher risk for postoperative complications.

These data may support informed discussion between surgeons and patients regarding the performance of SLNB in patients counseled for the surgical treatment of EIN. Given the paucity of data available, the objective of our study was to study the association of BMI with postoperative complications following SLNB during minimally invasive hysterectomy for EIN.

## 2. Materials and Methods

### 2.1. Data Collection

We used data from the National Surgical Quality Improvement Program (NSQIP) program database [21,22], which incorporates data from more than 700 hospitals across the US in 49 out of 50 states. The NSQIP database encompasses numerous variables that are collected prospectively, such as preoperative workup, operative time, and 30-day outcomes measures, such as surgical complications, readmissions, and more. Unlike administrative databases, the NSQIP offers detailed, prospectively collected, and clinically validated data, making it a powerful tool. It enables us to assess surgical outcomes, identify risk factors, and implement evidence-based strategies to reduce complications and enhance patient safety. Its comprehensive nature makes it a valuable resource for conducting multicenter studies and advancing evidence-based surgical care. The NSQIP website (https://www.facs.org/quality-programs/acs-nsqip, accessed on 1 March 2024) provides elaborate details on the process of data collection and regarding the definition of the various variables [23].

### 2.2. Study Population

The study cohort included women who underwent minimally invasive hysterectomy (laparoscopic or robotic-assisted) during January 2012–December 2020, with a postoperative histologic diagnosis of endometrial atypical hyperplasia or EIN; International Classification of Diseases (ICD)-9 codes 621.33 and 621.35 and ICD-10 code N85.02. We identified minimally invasive hysterectomy procedures with Current Procedural Terminology (CPT) codes 58570–58573. We excluded other types of hysterectomies, including abdominal, vaginal, radical, and supracervical hysterectomies. Laparoscopic-Assisted Vaginal Hysterectomy cases were excluded as well. For lymph node evaluation procedures, we excluded non-pelvic lymphadenectomies and cases with pelvic lymphadenectomy without concomitant SLNB-CPT codes 38500, 38510, 38525, 38562, and 38571–38573. We further excluded cases with preoperative sepsis, and non-elective surgeries. We excluded cases which lacked a complete recording of height and weight.

We categorized patients’ BMI according to the World Health Organization (WHO) classification: underweight (BMI < 18.5 kg/m^2^), normal BMI (BMI 18.5–24.9 kg/m^2^), overweight (BMI 25–29.9 kg/m^2^), obesity class 1 (BMI 30–34.9 kg/m^2^), obesity class 2 (BMI 35–39.9 kg/m^2^), and obesity class 3 (BMI ≥ 40 kg/m^2^).

### 2.3. Study Groups and Outcomes

We divided the study cohort into two groups, dichotomized by performance of SLNB yes/no, as per the CPT coding recorded. The primary outcome measures were complication rates in both study groups, stratified by BMI category. The secondary outcome measures were complication rates in the various BMI categories, only among women who underwent SLNB.

The study covariates included patient demographics, surgery information, and postoperative complications. Postoperative complications were defined using the Clavien–Dindo classification system [24]. Major complications included any of the following, occurring within 30 days of surgery: organ space surgical site infection, deep incisional surgical site infection, wound dehiscence, cerebrovascular accident, pulmonary embolism, deep venous thromboembolism, cardiac arrest, myocardial infarction, reoperation, or death. Minor postoperative complications included blood transfusion (within 72 h of surgery start time), superficial surgical site infection, urinary tract infection, acute renal insufficiency, and pneumonia.

### 2.4. Statistical Analysis

Study groups were compared through univariable analysis. For categorical variables we used the Chi-square test, and for continuous variables we used the Mann–Whitney U test. Categorical variables were reported as proportions and continuous variables as median [interquartile range].

Multivariable logistic binary regression analyses were performed to identify variables independently associated with postoperative complications. Receiver operating characteristic (ROC) analysis was performed to study the performance of BMI as a predictor of complications. All the statistical analyses were based on two-tailed hypotheses, and a *p* < 0.05 was considered statistically significant. IBM SPSS Statistics 29.0 was used for the statistical analyses. Figures were produced using GraphPad Prism version 6.0.0 for Windows, GraphPad Software, Boston, MA, USA.

### 2.5. Ethical Approval

The institutional review boards (IRBs) Office of Research Compliance and Quality Improvement reviewed and exempted this study (STUDY00003603).

## 3. Results

**Figure 1:**
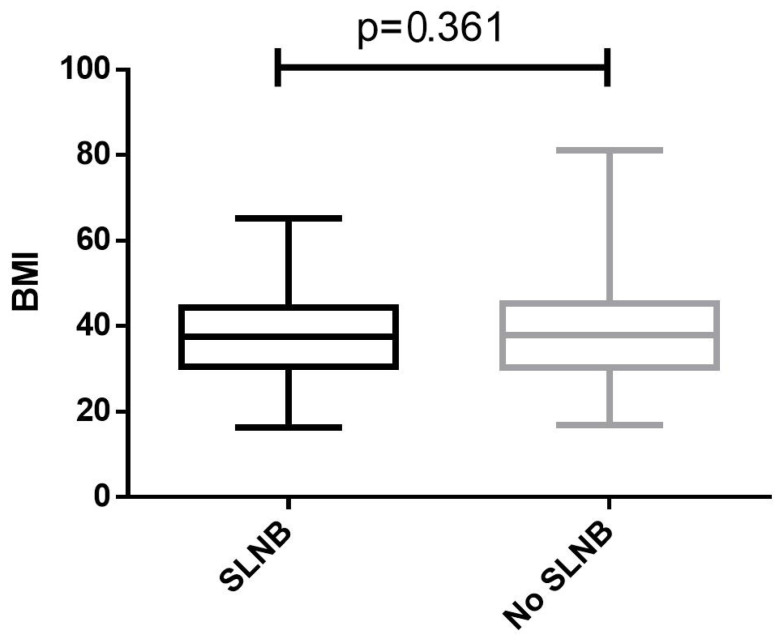
Body mass index parameters of study groups. BMI—body mass index; SLNB—sentinel lymph node biopsy.

**Figure 2:**
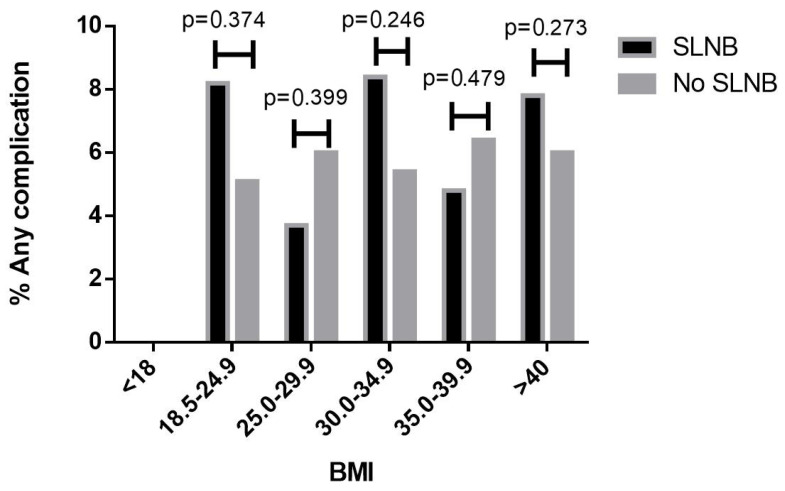
Rate of any complication of SLNB vs. no SLNB, stratified by body mass index categories.

A total of 4428 patients met the inclusion criteria. The median age was 56 [interquartile range (IQR) 49–63]. Of those, 584 (13.2%) had SLNB. The median BMI was 37.7 kg/m^2^ [IQR 30.4–45.2]. A total of 3389 (76.5%) patients were obese, and 1840 (41.6%) patients had BMI ≥ 40.0 kg/m^2^. The median surgical time was 124 min [IQR 95–159]. Patient characteristics are presented in Table S1. For the entire cohort, the rate of any complications was 6.0% (n = 264), major complications 2.3% (n = 101), and minor complications 4.2% (n = 187). BMI as a continuous variable, BMI categories, and the proportion of obese patients were similar among patients who underwent SLNB vs. those who did not (*p* = 0.361, *p* = 0.444, and *p* = 0.673, respectively, Table 1, Figure 1). When comparing the rate of any complications between patients who underwent SLNB vs. those who did not, stratified by BMI category, there was no association between SLNB performance and any complications in any of the BMI categories (Figure 2).

**Figure 3:**
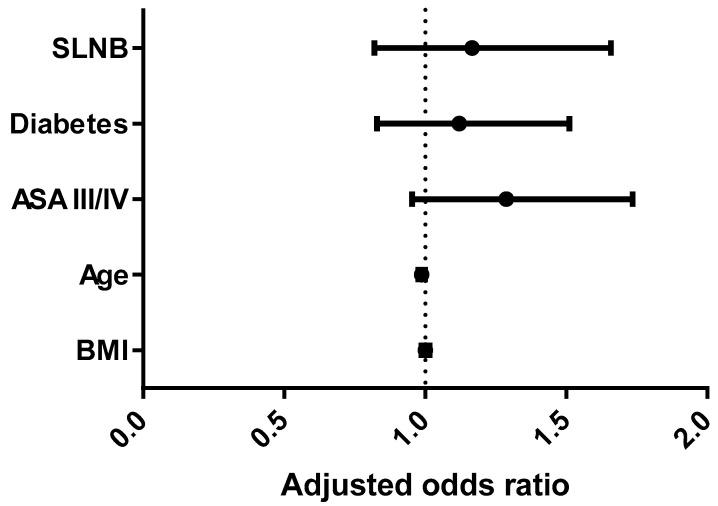
Multivariable binary regression analysis for predictors of any complication. ASA—American Society of Anesthesiologists.

In a multivariable binary regression analysis, BMI and the performance of SLNB were not independently associated with any complication [adjusted odds ratio (aORs) 1.001, 95% confidence interval (CI) (0.98–1.01), and aORs 1.1, 95% CI (0.82–1.65), respectively, Figure 3].

**Figure 4:**
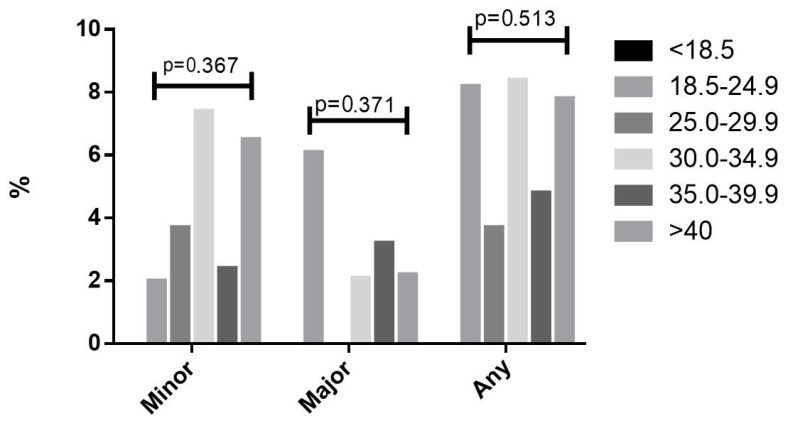
Complications in the sentinel lymph node group by body mass index categories.

**Figure 5:**
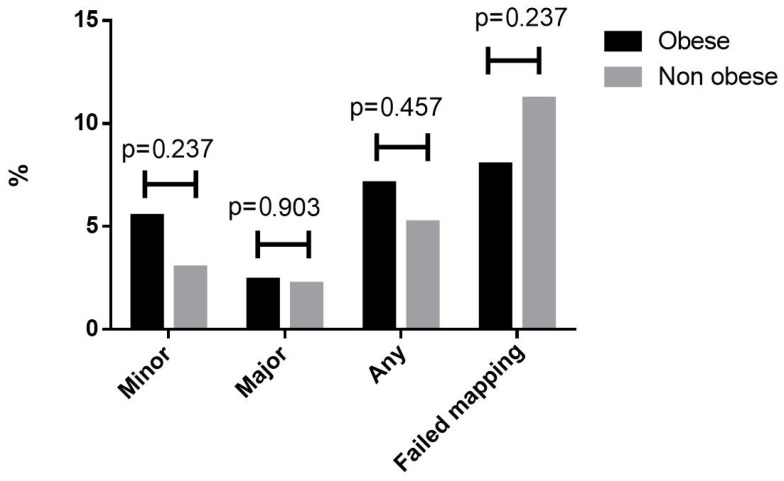
Complications in the sentinel lymph node group by obesity status.

In an analysis of the cohort of patients undergoing SLNB (n = 584), there was no association between the rates of any major or minor complications with BMI categories or obesity (Figure 4 and Figure 5). ROC analyses for the association between BMI and occurrence of any major or minor complications are presented in Figure S1a–c, and they show low performances. The rate of urinary tract infections was similar in obese (2.4%) and non-obese patients (1.8%), *p* = 0.244, and the rate of urinary tract infections was similar in the sub-analysis of the SLNB and no SLNB groups. The rate of superficial surgical infection was similar among the obese (1.4%) and non-obese (1.3%) groups, *p* = 0.741, and the rate of superficial surgical infection was similar in the sub-analysis of SLNB and no SLNB groups. The rate of deep surgical infection was also similar in the obese (1.1%) and non-obese groups (1.0%), *p* = 0.665. The rate of venous thromboembolic events was 0.2% in the obese and 0.2% in the non-obese groups, *p* = 0.918, and was similar in the SLNB and no SLNB groups. When examining BMI > 50, there was no difference in the rate of venous thromboembolic events; 0.3% in BMI ≥ 50 and 0.2% in BMI > 50, *p* = 0.338. The mean operative time was longer in the obese group, at 135 min ± 57 vs. 121 ± 47 min in the non-obese group (*p* < 0.001).

## 4. Discussion

### 4.1. Summary of Main Results

In this study of minimally invasive hysterectomies for EIN, 76.5% of patients were obese. SLNB and BMI were not associated with higher rates of complications, and these were similar in all BMI categories.

### 4.2. Results in the Context of the Published Literature

EIN presents a significant clinical concern due to its potential progression to endometrial carcinoma and due to its occurrence in patients with a high BMI. The surgical management of EIN typically involves hysterectomy, with or without lymph node assessment. There is growing evidence that SLNB is valuable in managing patients with EIN, specifically as approximately 50% of patients with EIN of preoperative biopsy eventually have a diagnosis of endometrial carcinoma [7]. However, currently, there are no established guidelines for lymph node evaluation in patients with EIN, leaving clinicians to depend on their professional judgment and experience. These results suggest concern over SLNB increasing surgical risk need not being an important factor in making that judgment.

The literature regarding the association of SLNB with postoperative complications in EIN obese patients is scarce. In fact, the literature regarding the association of BMI and SLNB in endometrial cancer is limited to mapping the failure rate of the SLN procedure [25], and the association between BMI, postoperative complications, and the SLNB procedure remains an unmet knowledge gap. In our results, both study groups had similar BMI parameters, indicating that elevated BMI did not seem to be a barrier to surgeons considering SLNB in minimally invasive hysterectomy for EIN.

The safety regarding adding SLNB to surgery for EIN is supported by the lack of an independent association between SLNB, BMI, and any postoperative complications in our cohort, which demonstrated low complication rates overall, ranging from 2.4 to 6.0%. The previous literature has underlined the safety of SLNB in endometrial cancer [26]. Interestingly, the inherent complications associated with lymph node evaluation, such as lymphocele and lymphedema, are not part of the routine definitions of postoperative complications in many population-based databases.

Although SLNB is generally considered safe, its addition inherently carries the risk of procedure-related complications, most notably lymphedema. Older studies in the literature report up to 40% of lymphedema in EC patients; however, that was before the era of SLNB [27].

A recent cost-analysis study underlined that performing lymph node SLNB for EI, without proceeding to full lymphadenectomy in cases of non-mapping, compared favorably to full lymph node dissection guided by the Mayo Criteria, and that in a population with a high prevalence of occult EC, consistently omitting SLNB leaves many patients inadequately staged [28]. Interestingly, that analysis included only robotic procedures, which may have higher costs than conventional laparoscopy if not used extensively.

We previously reported that SLNB performance in patients with EIN may increase the risk for venous thromboembolic events [11]. In this study, we did not find an association of BMI with venous thromboembolic events; however, it is possible that the power achieved with a larger sample size is needed to detect such an association. Within the sub-cohort of women who underwent SLNB, we did not find an association between BMI and any major or minor complications. This finding supports our finding from the primary outcome analysis that when SLNB is performed for EIN, patients with a higher BMI have no additional risk for any complications.

### 4.3. Strengths and Weaknesses

The strengths of our study include the large sample size, utilizing a national-level database in our analysis, and including data from years in which SLNB was performed for EIN. Moreover, we included only minimally invasive surgeries and EIN cases with strict inclusion and exclusion criteria. There are several limitations in this study. First, we could not account for preoperative diagnoses (EIN on preoperative biopsy). As up to 50% of patients with a preoperative diagnosis of EIN are ultimately diagnosed with endometrial cancer [3,4,5,6,7], this cohort underrepresents the actual number of patients with a preoperative diagnosis of EIN. However, the safety of SLNB for patients with endometrial cancer is already well established. In addition, we could not account for specific lymph node-related complications. Postoperative complications in the NSQIP database are limited to 30 days, and the negative impact of lymphedema on quality of life can be lifelong. Lastly, from this database we were not able to determine bias introduced by surgeon experience performing the SLNB (resident, fellow, gynecologic oncologist, or gynecologist), which could have narrowed differences in complication rates if a greater proportion of operations with SLNB were performed by experienced surgeons and more by trainees in surgeries without SLNB. The database analyzed does not differentiate between robotic-assisted procedures and conventional laparoscopies. Furthermore, long-term morbidity was not evaluated through our analysis, as numerous disorders emerge beyond the 30-day postoperative timeframe—this is a major limitation. The database lacks detailed information regarding surgeon experience, changes in surgical techniques, and intraoperative complications. Moreover, the absence of longitudinal follow-up constrains the evaluation of late-onset problems.

### 4.4. Implications for Practice and Future Research

These findings challenge the assumption that a higher BMI increases the risk of postoperative complications in minimally invasive hysterectomy for endometrial intraepithelial neoplasia. Surgeons can be reassured that obesity or a high BMI does not inherently elevate risk for complications when performing minimally invasive hysterectomies with or without sentinel lymph node biopsy for endometrial intraepithelial neoplasia.

## 5. Conclusions

We found no association between BMI and an increased risk of postoperative complications when performing SLNB during minimally invasive hysterectomy for EIN. Performing SLNB for EIN has a similar complication rate for all BMI categories.

## Figures and Tables

**Table 1:** Body mass index parameters of study groups.

Characteristic	Sentinel Lymph Node Biopsy (n = 584)	No Sentinel Lymph Node Biopsy (n = 3844)	*p* Value	Rate of SLNB
Body mass index, kg/m^2^	37.5 [30.5–44.2]	37.8 [30.3–45.4]	0.361	
Body mass index, kg/m^2^			0.444	
<18.5	2 (0.3%)	14 (0.4%)		12.5%
18.5–24.9	49 (8.4%)	373 (9.7%)		11.6%
25.0–29.9	82 (14.0%)	519 (13.5%)		13.6%
30.0–34.9	95 (16.3%)	626 (16.3%)		13.2%
35.0–39.9	126 (21.6%)	702 (18.3%)		15.2%
>40	230 (39.4%)	1610 (41.9%)		12.5%
Obese	451 (77.2%)	2938 (76.4%)	0.673	

Data are n (%) or median [interquartile range]; SLNB—sentinel lymph node biopsy.

## Data Availability

The data presented in this study are available on request from the corresponding author due to privacy restrictions.

## Supplementary Materials

The following supporting information can be downloaded at: https://www.mdpi.com/article/10.3390/cancers17081257/s1, Figure S1: Receiver operating curves for body mass index as predictor of complications; Table S1: Characteristics of the study cohort.

## Abbreviations

The following abbreviations are used in this manuscript:

EIN	Endometrial intraepithelial neoplasia
SLNB	Sentinel lymph node biopsy

## References

[1] Pennant M.E., Mehta R., Moody P., Hackett G., Prentice A., Sharp S.J., Lakshman R. (2017). Premenopausal abnormal uterine bleeding and risk of endometrial cancer. BJOG.

[2] van Hanegem N., Breijer M.C., Slockers S.A., Zafarmand M.H., Geomini P., Catshoek R., Pijnenborg J., van der Voet L., Dijkhuizen F., van Hoecke G. (2017). Diagnostic workup for postmenopausal bleeding: A randomised controlled trial. BJOG.

[3] Trimble C.L., Kauderer J., Zaino R., Silverberg S., Lim P.C., Burke J.J., Alberts D., Curtin J. (2006). Concurrent endometrial carcinoma in women with a biopsy diagnosis of atypical endometrial hyperplasia: A Gynecologic Oncology Group study. Cancer.

[4] Sherman M.E. (2000). Theories of endometrial carcinogenesis: A multidisciplinary approach. Mod. Pathol..

[5] Doherty M.T., Sanni O.B., Coleman H.G., Cardwell C.R., McCluggage W.G., Quinn D., Wylie J., McMenamin Ú.C. (2020). Concurrent and future risk of endometrial cancer in women with endometrial hyperplasia: A systematic review and meta-analysis. PLoS ONE.

[6] The American College of Obstetricians and Gynecologists (ACOG) (2023). Management of Endometrial Intraepithelial Neoplasia or Atypical Endometrial Hyperplasia: ACOG Clinical Consensus No. 5. Obstet. Gynecol..

[7] Rosati A., Vargiu V., Capozzi V.A., Giannarelli D., Palmieri E., Baroni A., Perrone E., Berretta R., Cosentino F., Scambia G. (2024). Concurrent endometrial cancer in atypical endometrial hyperplasia and the role of sentinel lymph nodes: Clinical insights from a multicenter experience. Int. J. Gynecol. Cancer.

[8] Matanes E., Amajoud Z., Kogan L., Mitric C., Ismail S., Raban O., Knigin D., Levin G., Bahoric B., Ferenczy A. (2023). Is sentinel lymph node assessment useful in patients with a preoperative diagnosis of endometrial intraepithelial neoplasia?. Gynecol. Oncol..

[9] Levin G., Matanes E., Brezinov Y., Ferenczy A., Pelmus M., Brodeur M.N., Salvador S., Lau S., Gotlieb W.H. (2024). Machine learning for prediction of concurrent endometrial carcinoma in patients diagnosed with endometrial intraepithelial neoplasia. Eur. J. Surg. Oncol..

[10] Levin G., Matanes E., Salvador S., Lau S., Gotlieb W. (2024). Time interval from biopsy of endometrial atypical hyperplasia to surgery and risk for concurrent endometrial carcinoma—A retrospective study. BJOG.

[11] Levin G., Wright J.D., Burke Y.Z., Hamilton K.M., Meyer R. (2024). Utilization and Surgical Outcomes of Sentinel Lymph Node Biopsy for Endometrial Intraepithelial Neoplasia. Obstet. Gynecol..

[12] Ricci E., Moroni S., Parazzini F., Surace M., Benzi G., Salerio B., Polverino G., Vecchia C.L.A. (2002). Risk factors for endometrial hyperplasia: Results from a case-control study. Int. J. Gynecol. Cancer.

[13] Linkov F., Edwards R., Balk J., Yurkovetsky Z., Stadterman B., Lokshin A., Taioli E. (2008). Endometrial hyperplasia, endometrial cancer and prevention: Gaps in existing research of modifiable risk factors. Eur. J. Cancer.

[14] Lim L.M., Erfani H., Furey K.B., Matsuo K., Guo X.M. (2024). Risks and benefits of sentinel lymph node evaluation in the management of endometrial intraepithelial neoplasia. Expert Rev. Anticancer Ther..

[15] Nicholson K., Macharia A., Furuya R., Manning C., Hacker M.R., Harris D.A., Esselen K., Dottino J. (2023). Association of body mass index with early age at diagnosis of endometrial intraepithelial neoplasia. Gynecol. Oncol..

[16] Ward Z.J., Bleich S.N., Cradock A.L., Barrett J.L., Giles C.M., Flax C., Long M.W., Gortmaker S.L. (2019). Projected U.S. State-Level Prevalence of Adult Obesity and Severe Obesity. N. Engl. J. Med..

[17] Boutari C., Mantzoros C.S. (2022). A 2022 update on the epidemiology of obesity and a call to action: As its twin COVID-19 pandemic appears to be receding, the obesity and dysmetabolism pandemic continues to rage on. Metabolism.

[18] Bouwman F., Smits A., Lopes A., Das N., Pollard A., Massuger L., Bekkers R., Galaal K. (2015). The impact of BMI on surgical complications and outcomes in endometrial cancer surgery—An institutional study and systematic review of the literature. Gynecol. Oncol..

[19] Gunderson C.C., Java J., Moore K.N., Walker J.L. (2014). The impact of obesity on surgical staging, complications, and survival with uterine cancer: A Gynecologic Oncology Group LAP2 ancillary data study. Gynecol. Oncol..

[20] Scribner D.R., Walker J.L., Johnson G.A., McMeekin D.S., Gold M.A., Mannel R.S. (2002). Laparoscopic pelvic and paraaortic lymph node dissection in the obese. Gynecol. Oncol..

[21] Sellers M.M., Merkow R.P., Halverson A., Hinami K., Kelz R.R., Bentrem D.J., Bilimoria K.Y. (2013). Validation of new readmission data in the American College of Surgeons National Surgical Quality Improvement Program. J. Am. Coll. Surg..

[22] Surgeons ACo (2015). User Guide for the 2014 ACS NSQIP Participant Use Data File (PUF). https://www.facs.org/media/hl0hfrxz/nsqip_puf_userguide_2014.pdf.

[23] Hicks-Courant K., Ko E.M., Matsuo K., Melamed A., Nasioudis D., Rauh-Hain J.A., Uppal S., Wright J.D., Ramirez P.T., Crean V. (2024). Secondary databases in gynecologic cancer research. Int. J. Gynecol. Cancer.

[24] Clavien P.A., Barkun J., de Oliveira M.L., Vauthey J.N., Dindo D., Schulick R.D., de Santibañes E., Pekoli J., Slankamenac K., Bassi C. (2009). The Clavien-Dindo classification of surgical complications: Five-year experience. Ann. Surg..

[25] Vargiu V., Rosati A., Capozzi V.A., Sozzi G., Gioè A., Berretta R., Chiantera V., Scambia G., Fanfani F., Cosentino F. (2022). Impact of Obesity on Sentinel Lymph Node Mapping in Patients with apparent Early-Stage Endometrial Cancer: The ObeLyX study. Gynecol. Oncol..

[26] Geppert B., Lönnerfors C., Bollino M., Persson J. (2018). Sentinel lymph node biopsy in endometrial cancer—Feasibility, safety and lymphatic complications. Gynecol. Oncol..

[27] Yost K.J., Cheville A.L., Al-Hilli M.M., Mariani A., Barrette B.A., McGree M.E., Weaver A.L., Dowdy S.C. (2014). Lymphedema after surgery for endometrial cancer: Prevalence, risk factors, and quality of life. Obstet. Gynecol..

[28] Walker A.R., Leite S., Taylor N., Graul A. (2025). Lymph node assessment at the time of robotic hysterectomy for endometrial intraepithelial neoplasia: A cost-effectiveness analysis. Gynecol. Oncol..

