# Supplementary material for: Examining the Association of Body Mass Index and Complications When Including Sentinel Lymph Node Biopsy in Minimally Invasive Surgery for Endometrial Intraepithelial Neoplasia
Source: Cancers (Basel). 2025 Apr 8;17(8):1257. doi: 10.3390/cancers17081257 (PMC12025501; doi:10.3390/cancers17081257)
Supplement: Supplementary file 1 [file cancers-17-01257-s001.zip › cancers-3529820-supplementary.pdf]

Table S1. Characteristics of the study cohort.

Characteristics	n=4,428 (%)
Sentinel lymph node biopsy	
Yes	584 (13.2%)
No	3,844 (86.8%)
**Demographics**	
Age, years	56 [49-63]
Body mass index, kg/m^2	37.7 [30.4-45.2]
Body mass index, kg/m^2	
≤18.5	16 (0.4%)
18.5-24.9	422 (9.5%)
25.0-29.9	601 (13.6%)
30.0-34.9	721 (16.3%)
35.0-39.9	828 (18.7%)
≥40	1,840 (41.6%)
Obese	
Yes	3,389 (76.5%)
No	1,039 (23.5%)
**Comorbidities**	
Diabetes	986 (22.3%)
Tobacco smoke	357 (8.1%)
Chronic obstructive pulmonary disease	54 (1.2%)
Chronic medications	2,226 (50.3%)
Corticosteroids use	67 (1.5%)
ASA III/IV	2,257 (51.0%)
**Outcomes**	
Surgical time, minutes	124 [95-159]
Hospital stay, days	1 [0-1]
**Complications**	
Major complication	101 (2.3%)
Minor complication	187 (4.2%)
Any complication	264 (6.0%)
Readmission	90 (2.0%)

ASA – American Society of Anesthesiologists

Data is n (%) or Median [Interquartile range]

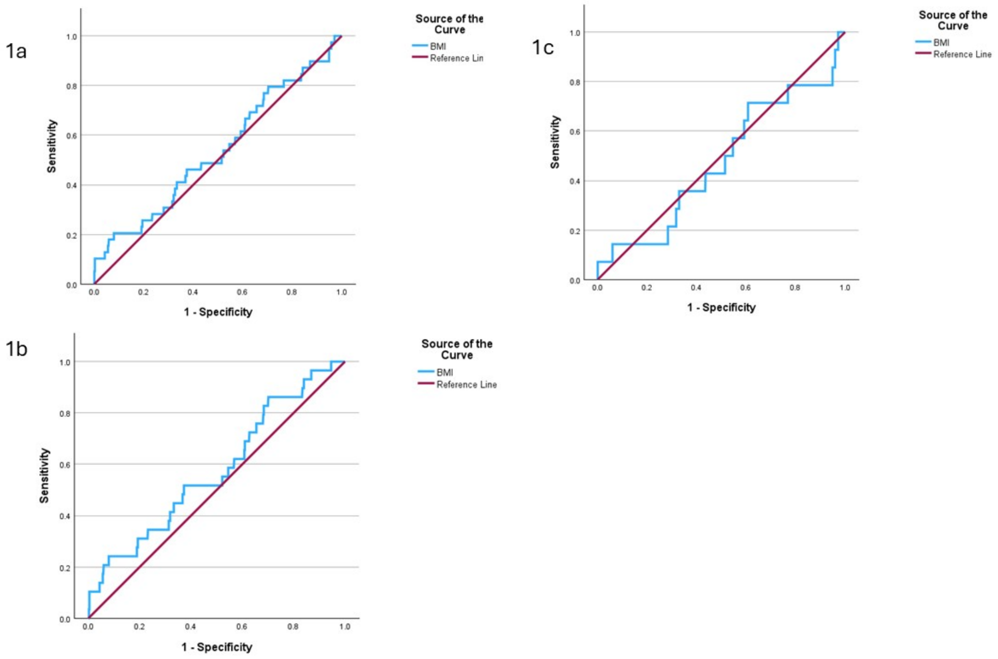

Figure S1. a-c. Receiver operating curves for body mass index as predictor of a. any complication, b. minor complication, c. major complication.
